# Comparative genome analysis of colistin-resistant OXA-48-producing *Klebsiellapneumoniae* clinical strains isolated from two Iranian hospitals

**DOI:** 10.1186/s12941-021-00479-y

**Published:** 2021-10-23

**Authors:** Negin Bolourchi, Fereshteh Shahcheraghi, Christian G. Giske, Shoeib Nematzadeh, Narjes Noori Goodarzi, Hamid Solgi, Farzad Badmasti

**Affiliations:** 1 Department of Bacteriology, Pasteur Institute of Iran, Tehran, Iran; 2 Division of Clinical Microbiology, Department of Laboratory Medicine, Karolinska Institutet and Karolinska University Hospital, Stockholm, Sweden; 3 Department of Pathobiology, School of Public Health, Tehran University of Medical Sciences, Tehran, Iran; 4 Department of Laboratory Medicine, Amin Hospital, Isfahan University of Medical Sciences, Isfahan, Iran

**Keywords:** Colistin-resistant *Klebsiella pneumoniae*, Carbapenemases producing strains, Hypervirulent plasmids

## Abstract

**Background:**

Carbapenemase-producing *Klebsiella pneumoniae* (CP-KP) is becoming extensively disseminated in Iranian medical centers. Colistin is among the few agents that retains its activity against CP-KP. However, the administration of colistin for treatment of carbapenem-resistant infections has increased resistance against this antibiotic. Therefore, the identification of genetic background of co-carbapenem, colistin-resistance *K.* *pneumoniae* (Co-CCRKp) is urgent for implementation of serious infection control strategies.

**Methods:**

Fourteen Co-CCRKp strains obtained from routine microbiological examinations were subjected to molecular analysis of antimicrobial resistance (AMR) using whole genome sequencing (WGS).

**Results:**

Nine of 14 *K.* *pneumoniae* strains belonged to sequence type (ST)-11 and 50% of the isolates had K-locus type 15. All strains carried *bla*_OXA-48_ except for P26. *bla*_NDM-1_ was detected in only two plasmids associated with P6 and P26 strains belonging to incompatibility (Inc) groups; IncFIB, IncHI1B and IncFII. No *bla*_KPC_, *bla*_VIM_ and *bla*_IMP_ were identified. Multi-drug resistant (MDR) conjugative plasmids were identified in strains P6, P31, P35, P38 and P40. MIC_colistin_ of *K. pneumoniae* strains ranged from 4 to 32 µg/ml. Modification of PmrA, PmrB, PhoQ, RamA and CrrB regulators as well as MgrB was identified as the mechanism of colistin resistance in our isolates. Single amino acid polymorphysims (SAPs) in PhoQ (D150G) and PmrB (R256G) were identified in all strains except for P35 and P38. CrrB was absent in P37 and modified in P7 (A200E). Insertion of IS*Kpn72* (P32), establishment of stop codon (Q30*) (P35 and P38), nucleotides deletion (P37), and amino acid substitution at position 28 were identified in MgrB (P33 and P42). None of the isolates were positive for plasmid-mediated colistin resistance (*mcr*) genes. P35 and P38 strains carried *iutA*, *iucD*, *iucC*, *iucB* and *iucA* genes and are considered as MDR-hypervirulent strains. P6, P7 and P43 had ICE*K*p4 variant and ICE*K*p3 was identified in 78% of the strains with specific carriage in ST11.

**Conclusion:**

In our study, different genetic modifications in chromosomal coding regions of some regulator genes resulted in phenotypic resistance to colistin. However, the extra-chromosomal colistin resistance through *mcr* genes was not detected. Continuous genomic investigations need to be conducted to accurately depict the status of colistin resistance in clinical settings.

**Supplementary Information:**

The online version contains supplementary material available at 10.1186/s12941-021-00479-y.

## Introduction

Carbapenemase-producing *Klebsiella pneumoniae* (CP-KP) has been established as a major cause of healthcare-associated infection in many geographic areas, with high morbidity and mortality [1]. Infection with CP-KP is a serious clinical problem because it is difficult to treat using conventional antibiotics. Resistance to carbapenems can be mediated by various mechanisms including the production of carbapenemases, including *K. pneumoniae* carbapenemase (KPC), New Delhi metallo-β-lactamase (NDM), and oxacillinase-48 (OXA-48), production of extended spectrum β-lactamases (ESBL) plus porins and hyperproduction of Ambler class C (AmpC) β-lactamase [2]. Among major carbapenemases, OXA-48 carbapenemase is currently the one that is spreading the most rapidly in the Middle East and other countries worldwide [3–5]. Polymyxins especially colistin are among the few agents that retain its activity against CP-KP, and they are considered as the key components against severe infections caused by these superbugs. Increasing administration of colistin for treatment of CP-KP infections has contributed to the emergence of acquired resistance against this antibiotic [6]. Resistance to colistin is mostly associated with LPS modification (result from mutations in *pmrA/pmrB*, *phoP/phoQ, mgrB, crrB*, and *ramA* genes) as well as overproduction of efflux-pumps (mediated by *kpnE/kpnF* and mutations of *acrB*) on the chromosomal level. Additionally, the production of phosphor-ethanolamine transferase encoded by plasmid-mediated colistin resistance (*mcr*) genes results in transferable colistin-resistance [7]. In Iran, where the carbapenemase is becoming extensively disseminated among clinical Gram-negative isolates, increasing rate of colistin-resistant *K. pneumoniae* is a critical matter. Therefore, the identification of genetic background of co-carbapenem, colistin-resistance *K. pneumoniae* (Co-CCRKp) is urgent for implementation of serious infection control strategies.

Genomic studies based on whole genome sequencing (WGS) accurately identify multi-drug resistant (MDR) and hypervirulent clones in outbreaks and provide data about diversity and antimicrobial resistance (AMR) reservoir of *K. pneumoniae* [8]. Furthermore, such studies are capable to determine the circulation of clinically important sequence types (ST) and spreading of major AMR genes across various clonal lineages and among hospitalized patients and community carriers [9]. Therefore, the global and regional awareness of antibiotic resistance genetic determinants is critical to combat the spread of highly resistant *K. pneumoniae* and decrease the number of victims.

In this study, we conducted a comparative genomic analysis of 14 colistin-resistant OXA-48-producing *K. pneumoniae* isolated from different wards of two Iranian hospitals with focus on their AMR genetic reservoir using WGS.

## Methods

### Bacterial isolates

In the period between January 2014 to March 2016, one hundred and thirty-eight carbapenem-resistance *K. pneumoniae* isolates were obtained from routine microbiological examinations on clinical samples (e.g., urine and bronchial aspirate) from two medical centers in two provinces of Iran (hospital A, a 496-bed university hospital, located in Tehran and hospital B, a 800-bed university hospital, located in Isfahan.). Conventional biochemical examinations were used for identification of the isolates. Genus and specie of isolates were confirmed by PCR-sequencing of 16s rRNA [10].

### Antimicrobial susceptibility testing

Antimicrobial susceptibility was determined using Kirby-Bauer disk diffusion method according to the clinical and laboratory standards institute (CLSI) guideline [11]. The minimal inhibitory concentration (MIC) of carbapenems (imipenem, meropenem and ertapenem) were determined by gradient test strips (Liofilchem, Italy). Broth microdilution method was utilized to determine the MIC of colistin using colistin sulfate (Merck, Germany). According to CLSI M100- S30, a MIC = 2 µg/ml was interpreted as intermediate susceptibility, whereas a MIC of ≥ 4 µg/ml was considered as resistance [11]. *E. coli* ATCC 25922 was used as a control strain for antimicrobial susceptibility testing.

### DNA extraction and whole genome sequencing

DNA extraction was performed using a bacterial genomic DNA kit (GenElute™, Sigma). Genome libraries were obtained from Illumina System (Illumina Inc., San Diego, CA).

### De novo assembly

The quality scores of the FASTQ paired-end files were checked using FastQC software [12]. Trimming and de novo assembly of short-read sequences was performed using Trimmomatic version 0.40 and SPAdes version 3.15.2, respectively [13] with k-mer = 99. The quality of contig assembly was assessed using QUAST software [14].

### Post de novo assembly

Genomic statistics of contig assemblies including genome length, GC content, N50 and number of coding sequences (CDSs), rRNA and tRNA as well as gene annotation were determined using DFAST (https://dfast.ddbj.nig.ac.jp/). Chromosomal and extra-chromosomal contigs were sorted using mlplasmids—version 1.0.0 (https://sarredondo.shinyapps.io/mlplasmids/). Multi-locus sequence typing (MLST) of chromosomal contigs was determined using MLST Server 2.0 (https://cge.cbs.dtu.dk/services/MLST/). Plasmid incompatibility (Inc) was checked using PlasmidFinder 2.0 server (http://cge.cbs.dtu.dk/services/PlasmidFinder/). Antibiotic resistance genes were detected using the comprehensive antibiotic resistance database (CARD) (https://card.mcmaster.ca/analyze/rgi). Capsular typing (K-typing), O typing and allelic determination were performed using the Kaptive webtool (https://kaptive-web.erc.monash.edu/). Taxonomic and phylogenetic analysis was performed using Type Strain Genome Server (https://tygs.dsmz.de/). Modification of phylogenetic tree was performed using the iTOL webtool (https://itol.embl.de). The structures of interactive conjugative element of *K. pneumoniae* (ICE*K*p) were characterized using NCBI BLAST (https://www.ncbi.nlm.nih.gov). Single amino acid polymorphisms (SAPs) of chromosomally-encoded proteins involved in AMR were identified using paired-wise alignment in NCBI P-BLAST.

## Results

### *K. pneumoniae* isolates and antimicrobial susceptibilities

Totally, 14 of 138 carbapenem-resistant *K. pneumoniae* isolates (10.1%) (P6, P7, P26, P31, P32, P33, P35, P36, P37, P38, P40, P41, P42, P43 and P44) showed colistin-resistant phenotype. All 14 strains were resistant to cefotaxime (CTX), ceftazidime (CAZ), cefepime (FEP), ciprofloxacin (CIP), ertapenem (ETP), meropenem (MEM) and imipenem (IMP). Strains were confirmed as colistin-resistant by broth microdilution method (according to 2020 CLSI M100- S30) with MICs ranged between 4 and 32 µg/ml. E-test results ranged from 0.5 to 8 µg/ml for ertapenem, 0.5 to 32 µg/ml for meropenem and 4 to 256 µg/ml for imipenem. Of 14 Co-CCRKp isolates, seven (50%) were isolated from intensive care unit (ICU), three from neurosurgery ward, two from emergency unit and two different isolates from a 55-year-old female outpatient. The strains were isolated from tracheal (7), urine (2), cerebrospinal fluid (2), blood (1), wound (1) and chest-tube (1). The clinical information of the strains is shown in Table 1.

**Table 1:** Clinical information of 14 colistin-resistant OXA-48-producing *K. pneumoniae* strains

Strain	Infection source	Hospital ward	Gender	Age	MIC Colistin
P6	Urine	Outpatient	Female	55	16
P7	Wound	Outpatient	Female	55	16
P26	Tracheal	ICU	Male	43	4
P31	Tracheal	ICU	Female	22	16
P32	Tracheal	ICU	Female	65	16
P33	Tracheal	Neurosurgery	Female	34	16
P35	Urine	ICU	Male	77	16
P36	Chest tube	ICU	Male	26	16
P37	Tracheal	ICU	Male	37	16
P38	CSF	ICU	Female	45	16
P40	Tracheal	Neurosurgery	Male	38	32
P42	Tracheal	Neurosurgery	Female	34	32
P43	CSF	Emergency	Male	57	32
P44	Blood	Emergency	Female	53	16

### Chromosomal characterization

#### Genetic features

The DFAST genetic analysis of purified contigs showed that chromosome length of 14 *K. pneumoniae* strains scaled from minimum 4,941,238 bp (for P38) to maximum 5,349,088 bp (for P37). The number of CDSs varried from 4694 to 5118. The highest N50 was calculated 215,119 belonging to P38. The GC content percentage for all isolated was 57%. The number of rRNA and tRNA varied from three to six and 51 to 80, respectively. See Additional file 1: Table S1.

#### Multilocus sequence typing (MLST)

**Fig. 1:**
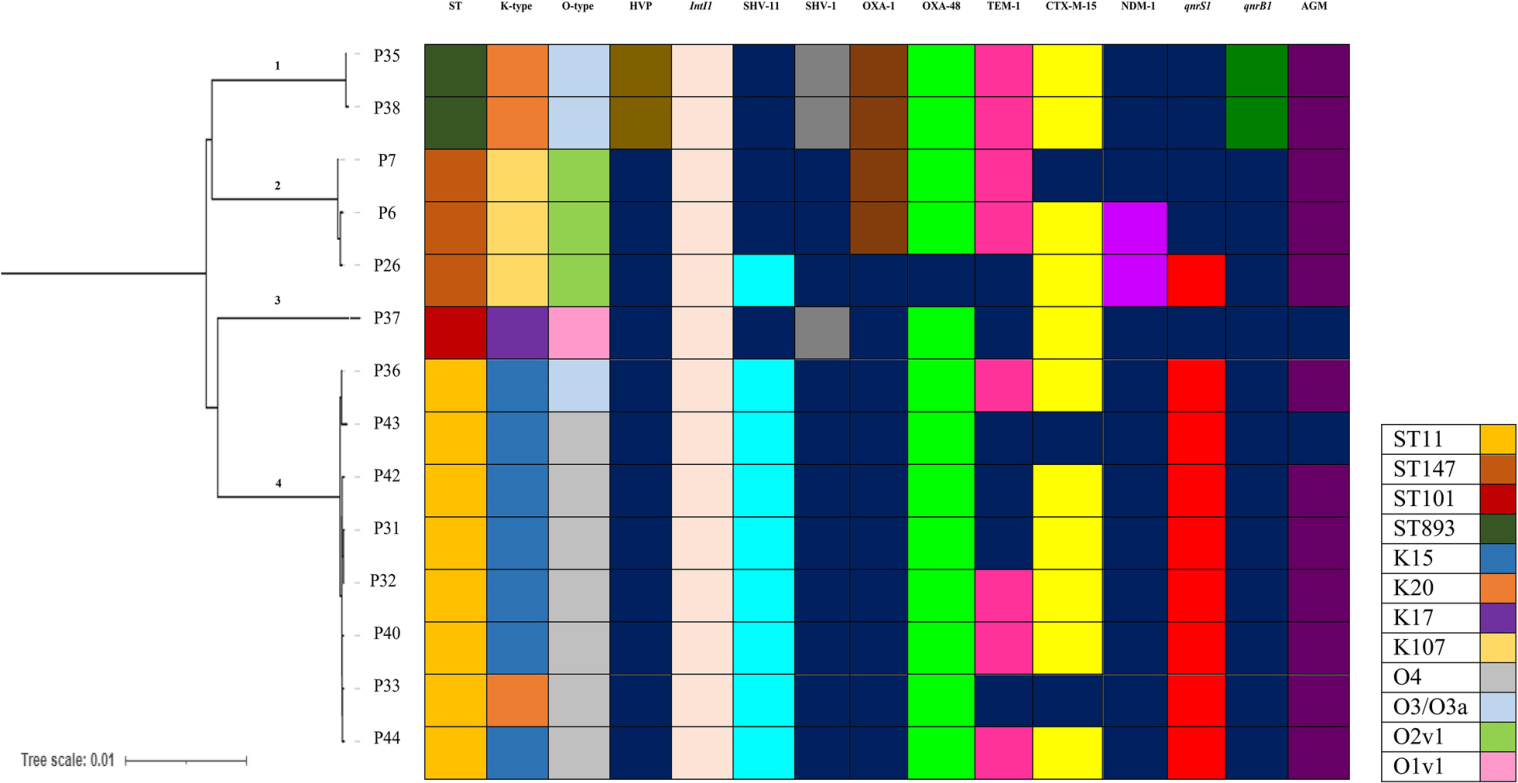
Phylogenetic tree and major genetic characteristics of 14 colistin-resistant OXA-48-producing *K. pneumoniae* strains including STs, serotypes, hypervirulence and major AMR genes. (HVP and AGM stand for hypervirulent plasmid and aminoglycoside modifying enzymes, respectively)

The MLST results showed that eight strains (57%) belonged to ST11 (P31, P32, P33, P36, P40, P42, P43 and P44), three strains belonged to ST147 (P6, P7 and P26), two strains belonged to ST893 (P35, P38) and one strains belonged to ST101 (P37). Figure 1. See Additional file 1: Table S1.

#### K-locus and O typing

The results of K-locus typing showed that P6, P7 and P26 had K107D1 (*wzc* 64 and *wzi* 64); P33, P35 and P38 had K20 (*wzc* 21 and *wzi* 150); P37 had K17 (*wzc* 18 and *wzi* 137) and the remained strains had K15 allelic profile (*wzc* 919 and *wzi* 50) (prevalence rate of 50%). Figure 1. In case of O-varient, P6, P7 and P26 belonged to O2v1 type; P35 and P38 belonged to O3/O3a; P37 belonged to O1v1 and P31, P32, P33, P36, P40, P42, P43 as well as P44 belonged to O4. See Fig. 1. See Additional file 1: Table S1.

#### AMR analysis

##### Resistance genes

The CARD results showed that our strains encoded AMR genes from different antibiotic classes including β-lactamases (*bla*_SHV_ family and *ampH*), ATP-binding cassette (ABC) antibiotic efflux pumps (*lptD* and *msbA*), major facilitator superfamily (*mfs*, *h-NS*, *emrR, tetB* and *tetR*), antibiotic efflux pumps (*kpnE*, *kpnG*, *kpnH* and *kpnG*), resistance-nodulation-cell division (RND) antibiotic efflux pumps (*oqxA* and *oqxB*, *marA*, *adeF*, *baeR*, *rsmA* and *crp*), *pmr* phosphoethanolamine transferase (*eptB* and *arnT*), bacterial porins (*OmpK37* and *OmpA*) and fosfomycin thiol transferase (*fosA6* and *fosA5*). See Additional file 1: Table S1.

##### Single amino acid polymorphisms (SAPs)

Single amino acid substitutions of chromosomally-encoded proteins involved in AMR were detected by comparing related coding regions of each strains with *K. pneumoniae* NTUH-K2044 (NC_012731) as the reference colistin-susceptible strain using paired-wise alignment. SAPs resulted in resistance to cephalosporins; PBP3, elfamycin; EF-Tu, fosfomycin; UhpT, fluoroquinolones; ParC, GyrA and GyrB and multiple drugs; MarR were identified. Table 2. All 14 strains underwent identical modifications in PBP3 (D350N, S357N), UhpT (E350Q) and EF-Tu (R234F). S3N (in P6, P7 and P26) and E86K (in P32, P33, P36, P37, P40, P42, P43 and P44) were detected for MarR protein. MarR was found intact in strains P31, P35 and P38. See Table 2. S80I was detected for ParC in all strains except for P38 (P403A). GyrA was modified identically in all strains (S83I) except for P37 and P43 (D87G). GyrB was found intact in all strains except for P6, P7, P26 and P43 (E466D). Regarding colistin-resistance; *mgrB* was found intact in P6, P7, P26, P31, P36, P40, P43 and P44. However, P35 and P38 underwent a stop codon (Q30*) along MgrB protein sequence. Alteration of MgrB in P32 was mediated by insertion of IS*Kpn72*. Moreover, P37 had a large deletion and frame shift in the *mgrB* coding region. Amino acid modification (C28W) of MgrB was detected in P33 and P42. SAP of PhoQ (D150G) was identified in all strains except for P35 and P38. No amino acid alteration was found related to PhoP and AcrB. Amino acid substitutions of PmrA were detected in P35 and P38 (A41T) and P37 (A218V). SAP in PmrB (R256G) was identified in all strains except for P35, P37 and P38. RamA underwent modifications in P6, P7, P26 (D71B) and P43 (I25T). See Table 2. Modification of CrrB was only detected in P7 (A200E). Interestingly, CrrB was absent in P37.

**Table 2:** Single amino acid polymorphisms related to antimicrobial resistance in 14 colistin-resistant OXA-48-producing *K. pneumoniae* strains

Protein	Strains														
	Cephalosporin resistance	Elfamycin resistance	Fosfomycin resistance	Repressor of multi-drug resistance operon	Fluoroquinolone resistance			Colistin resistance							
	PBP3	EF-Tu	UhpT	MarR	ParC	GyrA	GyrB	PhoP	PhoQ	PmrA	PmrB	MgrB	RamA	AcrB	CrrB
P6	D350N, S357N	R234F	E350Q	S3N	S80I	S83I	E466D	Intact	D150G	Intact	R256G	Intact	D71B	Intact	Intact
P7	D350N, S357N	R234F	E350Q	S3N	S80I	S83I	E466D	Intact	D150G	Intact	R256G	Intact	D71B	Intact	A200E
P26	D350N, S357N	R234F	E350Q	S3N	S80I	S83I	E466D	Intact	D150G	Intact	R256G	Intact	D71B	Intact	Intact
P31	D350N, S357N	R234F	E350Q	Intact	S80I	S83I	Intact	Intact	D150G	Intact	R256G	Intact	Intact	Intact	Intact
P32	D350N, S357N	R234F	E350Q	E86K	S80I	S83I	Intact	Intact	D150G	Intact	R256G	Insertion of IS*Kpn72*	Intact	Intact	Intact
P33	D350N, S357N	R234F	E350Q	E86K	S80I	S83I	Intact	Intact	D150G	Intact	R256G	C28W	Intact	Intact	Intact
P35	D350N, S357N	R234F	E350Q	Intact	S80I	S83F	Intact	Intact	Intact	A41T	Intact	Stop codon (Q30*)	Intact	Intact	Intact
P36	D350N, S357N	R234F	E350Q	E86K	S80I	S83I	Intact	Intact	D150G	Intact	R256G	Intact	Intact	Intact	Intact
P37	D350N, S357N	R234F	E350Q	E86K	S80I	D87N	Intact	Intact	D150G	A218V	Intact	Deletion	Intact	Intact	ND
P38	D350N, S357N	R234F	E350Q	Intact	P403A	S83F	Intact	Intact	Intact	A41T	Intact	Stop codon (Q30*)	Intact	Intact	Intact
P40	D350N, S357N	R234F	E350Q	E86K	S80I	S83I	Intact	Intact	D150G	Intact	R256G	Intact	Intact	Intact	Intact
P42	D350N, S357N	R234F	E350Q	E86K	S80I	S83I	Intact	Intact	D150G	Intact	R256G	C28W	Intact	Intact	Intact
P43	D350N, S357N	R234F	E350Q	E86K	S80I	D87G	E466D	Intact	D150G	Intact	R256G	Intact	I25T	Intact	Intact
P44	D350N, S357N	R234F	E350Q	E86K	S80I	S83I	Intact	Intact	D150G	Intact	R256G	Intact	intact	Intact	Intact

*ND* means not detected

#### Phylogenetic analysis

The genome-based phylogenteic tree showed that our 14 Co-CCRKp starins are devided to four clades as follows: P35 and P38 = clade 1; P6,P7 and P26 = clade 2; P37 = clade 3 and P31, P32, P33, P36, P40, P42, P43 and P44 = clade 4. The dendrogram demonstrated that the phylogenetic categorization of the strains almost matched with their classification based on ST, K-type and O-type. See Fig. 1.

#### Integrative conjugative element (ICE*K*p)

**Fig. 2:**
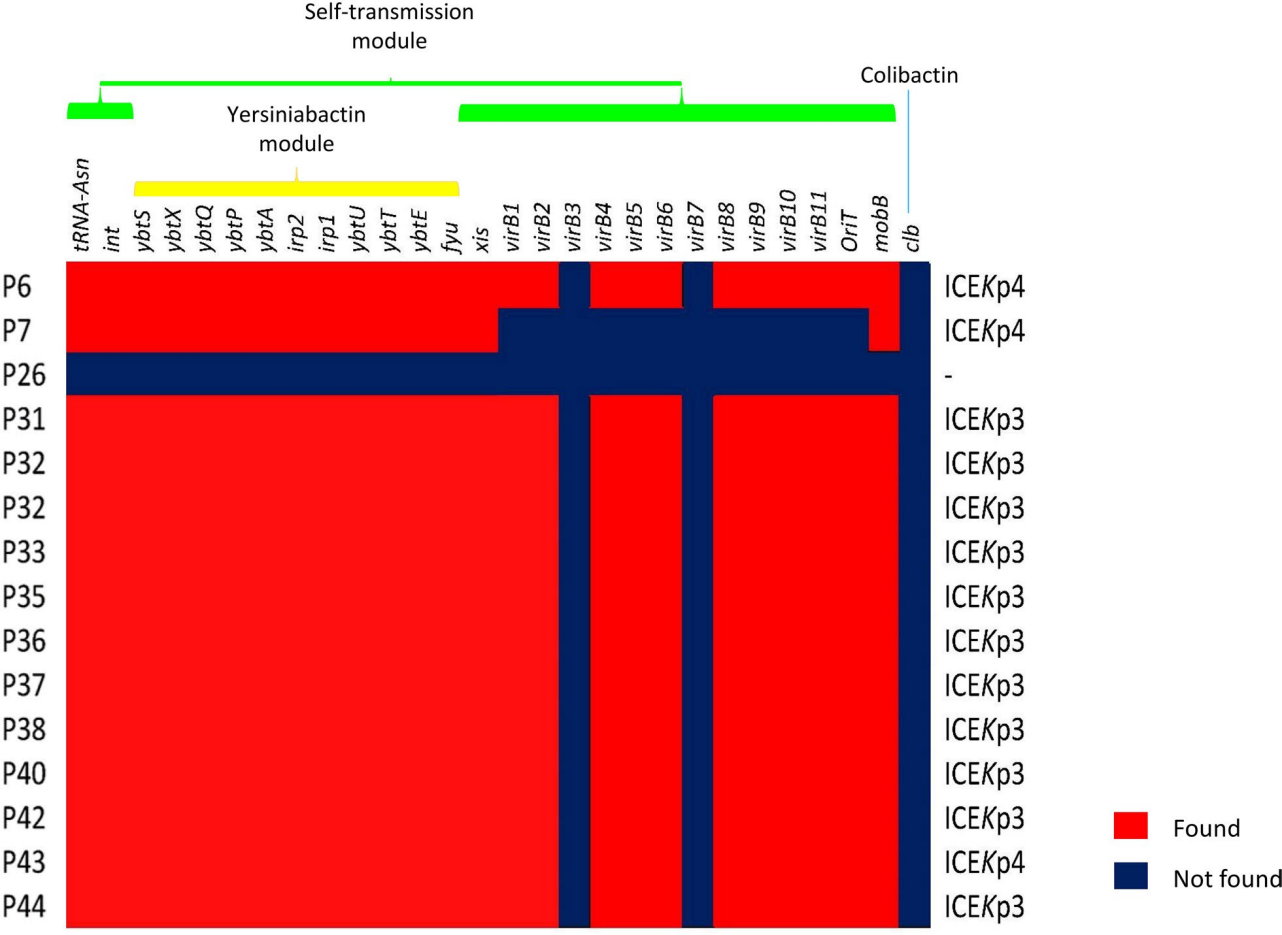
The interactive conjugative element structures of 14 colistin-resistant OXA-48-producing *K. pneumoniae* strains. Two main modules including yersiniabactin (*ybtS, ybtX, ybtQ, ybtP, ybtA, irp2, irp1, ybtU, ybtT, ybtE, and fyuA*) and self-mobilization (*int, xis*, *virB1*, *virB2*, *virB4*, *virB5*, *virB6*, *virB8*, *virB9*, *virB10*, *virB11*, *mobB* genes and *oriT* site) were identified and no colibactin was detected. ICE*K*p3 was the prodominant ICE*K*p variant among 13 positive strains

Two distinct modules were identified in ICE*K*p structures of our strains: Yersiniabactin (*ybtS, ybtX, ybtQ, ybtP, ybtA, irp2, irp1, ybtU, ybtT, ybtE* and *fyuA*) and self-mobilization (*int, xis*, *virB1*, *virB2*, *virB4*, *virB5*, *virB6*, *virB8*, *virB9*, *virB10*, *virB11* and *mobB* genes as well as *oriT* site). No colibactin was identified. ICE*K*p structures were detected in all 14 strains except for P23. P6, P7 and P43 carried ICE*K*p4 and the rest of the strains (78%) harbored ICE*K*p3. The *virB3* and *virB7* genes were absent in all ICE*K*p structures. The locus of *virB* was not identified in ICE*K*p of P7. See Fig. 2.

### Extra-chromosomal characterization

#### Genetic features

**Fig. 3:**
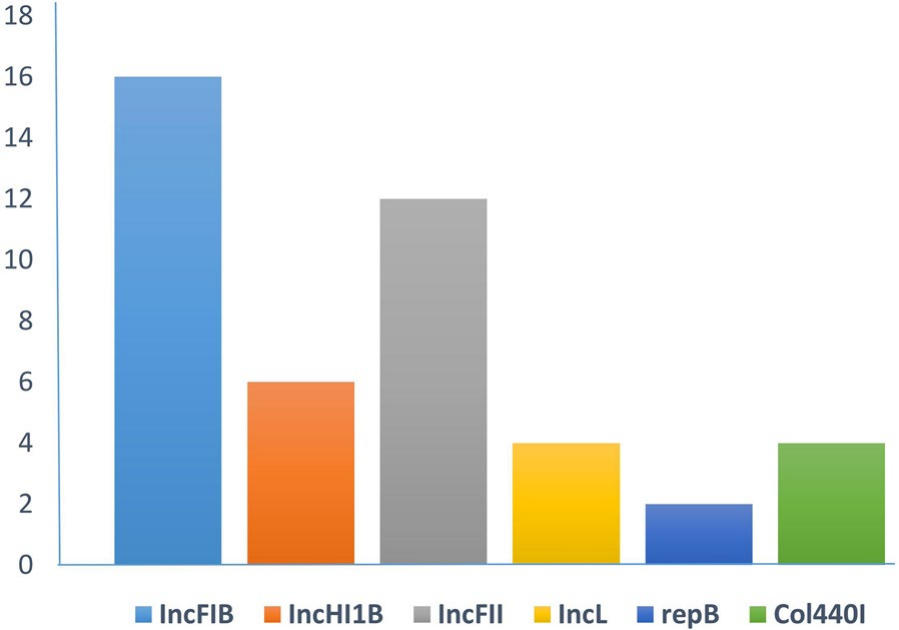
Compared frequencies of different plasmid incompatibility (Inc) types among 14 colistin-resistant OXA-48-producing *K. pneumoniae* strains. The results showed that the majority of plasmids belonged to IncFIB and IncFII

Thirty-five putative plasmids were assembled. Strains P6, P35, P37, P38, P40 and P44 carried three plasmids and P7, P26, P31, P32, P33, P36, P42 and P43 carried two plasmids. Plasmids incompatibility (Inc) groups were identified as IncFIB = 16, IncFII = 12, IncHI1B = 6, Col440I = 4, IncL = 4 and repB = 2. See Fig. 3. One/two plasmids in P6, P7, P35, P38, P40 and P44 strains were positive for all four compartment of relaxsasome complex including *oriT*, relaxsase, type IV secretion system (T4SS) and type IV coupling protein (T4CP). P31, P32, P36 and P42 carried one plasmid lacking only T4CP. Integron class 1 was detected in plasmids of all 14 K*. pneumoniae* strains*.* See Additional file 2: Table S2.

#### AMR and hypervirulence analysis

AMR genes related to multiple antibiotic drug classes were identified in majority (85%) of plasmids. ESBLs including *bla*_OXA-48_, *bla*_OXA-1_, *bla*_CTX-M-15_, *bla*_TEM-1_, and *bla*_NDM-1_ were identified. Figure 1. *bla*_OXA-48_ was detected in all Co-CCRKp isolates, with the exception of P26. Strains P6, P7, P35 and P38 encoded *bla*_OXA-1_. *bla*_CTX-M-15_ and *bla*_TEM-1_ were identified in 78% and 57% of the strains, respectively. The *bla*_NDM-1_ gene was found in only two plasmids related to strains P6 and P26 (both belonging to ST147). No *bla*_KPC_, *bla*_VIM_ and *bla*_IMP_ were identified. AMR genes related to aminoglycoside modifying enzymes including *aph(3'')-Ib*, *aac(6')-Ib9*, *aph(6)-Id*, *aac(6')-Ib-cr6 aadA5*, *rmtC* and *rmtF* were found in all strains except for P37 and P43. *qnrS1* was detected in 71% of the strains. *qnrB1* was identified only in plasmids related to P35 and P38. See Fig. 1. Other AMR genes were *tetB*, *tetR*, *ermB*, *arr2*, *mphA*, *catB3* and *dfrA14*. No *mcr* was detected. Strains P35 and P38 carried *iutA*, *iucD*, *iucC*, *iucB* and *iucA* hypervirulence genes on their plasmids. See Additional file 2: Table S2.

#### The *bla*_OXA-48_ and *bla*_NDM-1_ genetic environment

The gene annotation of OXA-48-producing plasmids showed that *bla*_OXA-48_ was flanked upstream/downstream by the LysR family transcriptional factor (*lysR*) in almost all strains. Additional file 3: Figure S1A. In P31, *bla*_OXA-48_ was flanked downstream by insertion sequence (IS) element transposase and *bla*_CTX-M-15_. In P32, two *bla*_OXA-48_ genes were identified on two distinc regions which environmet for one of them was similar to P31. The latter was flanked upstream by IS*6* family transposase. In P33, the *lysR* and *bla*_OXA-48_ complex was flanked downstream by three hypotetical genes and a transposase. The *lysR* and *bla*_OXA-48_ complex, *dhps*, *aph(6)-Id* and *bla*_CTX-M-15_ were surrounded by IS*4* and a transposase element in P35. In P36, P37, P38 and P44, the *lysR* and *bla*_OXA-48_ complex was flanked upstream by IS*91*, IS*4* and IS*110* family transposase genes, respectively. No transposase element was found surrounding the *lysR* and *bla*_OXA-48_ complex in P6, P7, P40, P42 and P43. *bla*_NDM-1_ was flanked upsteram by bleomycin binding protein and *trpF* genes and downstream by IS*91* family transposase in both P6 and P26. See Additional file 3: Figure S1B.

## Discussion

Our understanding about the genetic background of *K. pneumoniae* has increased worldwide. However, few WGS-based studies have focused on clinical *K. pneumoniae* isolates in Iran and consequently; the detalied genomic knowledge of cirulating STs in clinical settings is still limited in our country. WGS data for 14 clinical Co-CCRKp strains isolated between 2014 and 2016 in Iran were analyzed to provide a comparative genetic background that will help us to upgarde our information regarding epidemiology of AMR determinants.

One of the most clinically important AMR genes in *K. pneumoniae* isolates is class D β-lactamase *bla*_OXA-48_ [15]. The *bla*_OXA-48_ gene has been carrying by various plasmids Inc types including IncL/M, IncN, and IncA/C [16]. However, the results of our plasmid replicon typing shows that *bla*_OXA-48_ is carried by various incompatibility groups including IncL, Col440I, IncFIB and mainly IncFII. The genetic analysis of *bla*_OXA-48_ environment in our strains indicates that the LysR family transcriptional factor gene is closely related to *bla*_OXA-48_ carriage in *K. pneumoniae*. Detection of *bla*_OXA-48_ in transferrable plasmids is a clinical emergency as it can rapidly be widespread among other strains and even other *Enterobacterales* mediating highly resistant outbreaks in healthcare settings. P6, P31, P35, P38 and P40 are considered clinically significant strains as they contain conjutaive plasmids carrying *bla*_OXA-48_ carbapenemase. This suggests that carbapenem-resistance soon will disseminate in ICU and other wards among Iranian medical settings. Also, the carriage of a conjugative OXA-48-producing plasmids in an outpatient is highly troublesome as it implies the higher prevalence rates of carbapenemase in the community in near future. In the presented study, we reported the first extra-chromosomal carriage of two *bla*_OXA-48_ in strain P32. The double carriage of *bla*_OXA_ family by a single strain is rarely reported and is a matter of concern due to more rapid transmission of this significant carbapenemase. Sherchan and colleagues reported two copies of *bla*_OXA-181_ on ST147 *K. pneumoniae* from Nepal in 2020 which was previously reported from Pakistan, the United Arab Emirates and Korea [17].

P6 and P26 strains were detected as the only NDM-1 producing strains in our study indicating lower rate of *bla*_NDM-1_ carriage compared to other β-lactamase such as *bla*_CTX-M-15_ and *bla*_TEM-1_. However, higher prevalence of *bla*_NDM-1_ was reported form other Middle Eastern countries specifically Egypt and Saudi Arabia [18, 19]. In the study by Ghaith et al*.* [20] 52.2% of *K. pneumoniae* strains isolated from neonatal ICU in Cairo were NDM-1 producers. The prevalence of *bla*_NDM-1_ among *Enterobacterales* is mediated by rapid dissemination of conjugative plasmids [16]. In our study, none of the NDM-1 producing plasmids were conjugative. *bla*_NDM–1_ gene has been detected on plasmids of various incompatibility groups including IncF, IncA/C, IncL/M, IncH, IncN, and IncX3 or untypeable [16]. However, both of NDM-1 producing plasmids in our study belonged to IncFIB and IncHI1B. Co-carriage of *bla*_NDM-1_ and *bla*_OXA-48_ genes was seen in strain P6. Solgi et al. investigated the ESBLs carriage of 71 clinical carbapenem-resistant *Enterobacterales* in an Iranian hospital in Tehran. In this study, among 62 bacterial isolates, 46% and 37% of the isolates harbored *bla*_NDM-1_ and *bla*_OXA-48_, respectively and co-carriage of *bla*_NDM-1_ and *bla*_OXA-48_ was detected in 16% of the isolates. Also, the plasmid incompatibility types IncFII and IncA/C were identified among the NDM-1 producing isolates, while only IncL/M was detected among OXA-48 producers [16]. Co-existence of the *bla*_NDM-1_ and *bla*_OXA-48_ genes among CR-KP isolates was previously reported from other Middle Eastern countries such as Egypt. In a recent study by El-Domany and colleagues [21], 50 isolates co-carrying *bla*_NDM-1_ and *bla*_OXA-48_ were reported among 230 *K. pneumoniae* clinical isolates. In this study, the rate of *bla*_NDM-1_ and *bla*_OXA-48_ was reported 70.0% and 52.0%, respectively. In our study, ST11, ST147, ST893 and ST101 were *bla*_OXA-48_ and *bla*_NDM-1_ carriers in agreement with previous reports of OXA-48 and NDM-1 producing *K. pneumoniae* from Iran [16, 22]. These consensus reports suggest successful circulation of mentioned STs in Iran. The clonal carriage of *bla*_OXA-48_ and *bla*_NDM-1_ among *K. pneumoniae* strains may be region-specific in a single geographical territory despite high burden of individual transits. Accordingly, a significant association was reported for ST199 and ST152 with *bla*_OXA-48_ and *bla*_NDM-1_ carriage form Saudi Arabia, respectively [23, 24].

Co-existance of *bla*_OXA-48_, *bla*_CTX-M-15_ and *bla*_TEM-1_ within one single plasmid was observed in P6, P31, P32, P35, P38 and P44. However, this complex production was also observed in separate plasmids of P7, P40 and P42. Production of OXA-48 in *K. pneumoniae* plasmids seems to be typically concurrent with other ESBLs mainly *bla*_CTX-M_ and *bla*_TEM._ In one study*,* 88 of 94 of *K. pneumoniae* isolates in an Iranian hospital harbored *bla*_SHV,_ *bla*_CTX-M-15_ and *bla*_TEM-1_ concurrently while only one and two isolates solely carried *bla*_CTX-M-15_ and *bla*_SHV_, respectively [15]. The transmission of major ESBLs among *K. pneumoniae* has been reported in conjugative IncL/M and IncFII plasmids with ability of interspecies transfer [23, 25]. Accordingly, in our extra-chromosomal analysis, IncFII was a major plasmid replicon type and IncL was detected in two conjugative ESBL producer plasmids.

Resistance to colistin has been increasingly reported in the world including the Middle East region. A high resistance rate of 16.9% was reported between 2015 and 2016 from Iran [26]. Totally, 524 colistin-resistant *K. pneumoniae* isolates were reported from Turkey and Iran between 2013 and 2018 [27]. Jafari et al*. *[28] reported an increase of colistin resistance up to 50% in carbapenem-resistant *K. pneumoniae* isolates.

In the presented study; amino acid substitution, premature termination, deletion and insertion of IS element were identified in the coding region of MgrB as well as PmrA/PmrB, PhoP/PhoQ, RamA and CrrB regulators. MgrB inactivation was identified in 42% of the strains including P32, P33, P35, P37, P38 and P42. Substitution of cysteine at position 28 of MgrB with tryptophan was detected in P33 and P42. Position 28 is considered as an important region for amino acid substitution due to the key role of disulfide bonds of cysteine residue in MgrB functionality [29]. Similar SAPs such as C28F, C28Y and C28S has been previously reported in several different studies [6, 30]. In addition, truncation of MgrB at position 30 (Q30*) was identified in P35 and P38. Position 30 is highly prone to be modified in long exposure to colistin and therefore; (Q30*) has been commonly reported in other studies [29]. Insertional inactivation of MgrB was identified in only one strain (P32) mediating by insertion of IS*Kpn72* element (belonging to IS*4* family) in coding region of MgrB. Zhang et al. recently demonstrated the phenotypic switch of colistin-susceptible *K. pneumoniae* strains to colistin-resistant ones by horizontal transfer of an IS*Kpn72* carrying-plasmid. Therefore, in spite of chromosomal origin of MgrB; its modification can be plasmid-mediated and depended on conjugation processes. However, this phenomenon is not probable in our study because both plasmids of P32 were non-conjugative [31]. In this study, *crrB* gene was not carried by the P37 chromosome. The absence of *crrB* was previously reported in the study by Jayol et al*.*[32] which may be due to the differences in the lateral acquisition of the *crrAB* operon in *K. pneumoniae*. Thomas et al. [33] claimed that the lack of CrrB leads to significantly higher MIC_colistin_ and bacterial virulence than MgrB disruption in CR-KP. However, it seems that such manifestation cannot be attributed to P37. SAPs in PhoQ and PmrB were detected in 85% and 78% of the strains, respectively. PhoQ and PmrB modifications have been frequently reported in colistin-resistant *K. pneumoniae* isolates. In the study by Haeili et al*. *[26] 95% of 20 *K. pneumoniae* strains underwent point mutations in PmrB of which R256G was detected in five strains. However, the amino acid substitution in PhoQ or PmrB does not always correspond colistin-resistance [30, 34, 35]. Zhu et al*.* [36] reported mutated PhoQ in seven colistin-susceptible isolates of *K. pneumoniae*. The authors also suggested that the exposure of the isolates to colistin during treatment resulted in new PhoQ alterations mediating resistance to colistin. It is evident that upregulation of *pmrHFIJKLM*, *pmrCAB*, as well as *pmrK* operons is associated with colistin resistance in *K. pneumoniae* [37, 38]. Therefore, the mechanism of colistin resistance needs to be carefully interpreted.

The prevalence of hypervirulence among MDR *K. pneumoniae* is increasing worldwide [39]. In a meta-analysis conducted by Sanikhani et al*. *[40] the rate of reported hypervirulent *K. pneumoniae* isolates was determined 21.7% globally which majority of them were from China. In our study, P35 and P38 carried non-conjugative hypervirulence plasmids belonging to repB plasmid replicon type and considered as MDR-hypervirulent strains. This may suggest that P35 and P38 possess hypervirulent background which acquired MDR plasmids through conjugative processes. The rate of hypervirulence was 14% in our study and both strains were positive for *iutA*, *iucD*, *iucC*, *iucB* and *iucA*. Few studies have reported hypervirulent *K. pneumoniae* in Iran. Pajand et al. investigated the presence of hypervirulence genes in *K. pneumoniae* clinical isolates. *peg344*, *iucA*, *rmpA*, *rmpA2*, *iroB1*, *iroB2* and *iutA* were detected among all carbapenem-resistant isolates with 1.8%, 1.8%, 1.8%, 1.8%, 7.3%, 12.7% and 18.2% rate of carriage, respectively. The results of their study indicated that the prevalence of *iutA* was even higher in NDM-1 producing isolates [41]. Taraghian et al*.* detected 11 MDR-hypervirulent strains among 105 urinary tract *K. pneumoniae* isolates. In their study, ESBLs were identified in all hypervirulent strains and their carriage by hypervirulent stains were significantly higher compared to the classical ones [42]. In addition, Sanikhani et al. [43] reported the prevalence rate of 21.38% among 477 *K. pneumoniae* clinical isolates with high percentage of MDR and high-level resistance to imipenem.

The ICE*K*p analysis highlighted that the majority of our strains (78%) carried ICE*K*p3. According to an investigation by Lam et al*.* among 2498 *K. pneumoniae* genomes belonging to 37 different STs; ICE*K*p4, ICE*K*p3, ICE*K*p10 and ICE*K*p5 were the most common types of ICE*K*p. The results of this study also revealed that ICE*K*p variants can be integrated more frequently in specific STs compared to the other ones. The authors showed that almost 40% of the ICE*K*p positive *K. pneumoniae* strains belonged to CC258 [44]. Similarly, another study from South America indicated that the rate of ICE*K*p carriage is higher in strains belonging to ST11 and ST340. In our study, almost all ST11 strains harbored ICE*K*p3. This specific carriage of ICE*K*p3 in *K. pneumoniae* ST11 was previously reported by a Turkish study in 2019 [45].

## Conclusion

In this study, we assembled the whole-genomes of 14 colistin-resistant OXA-48-producing *K. pneumoniae* referred to two Iranian hospitals as well as their plasmids. The prevalence of *bla*_OXA-48_ in our strains was very high as well as *bla*_SHV,_ *bla*_CTX-M-15_ and *bla*_TEM-1_. MDR conjugative plasmids as well as hypervirulent plasmids were detected in some strains. Amino acid substitution, premature termination, deletion and insertion of an IS element were identified in the coding region of MgrB. SAPs in PmrA, PmrB, PhoQ, RamA and CrrB regulators were involved in colistin resistance. Pan-genomic investigations can provide data on AMR reservoir of highly-resistant *K. pneumoniae* at clinical and even community levels and ultimately provide a realistic status of AMR countrywide.

## Supplementary Information

**Additional file 1: Table S1.** Genotypic, resistome profile and capsular and O-typing of 14 colistin-resistant OXA-48-producing *K. pneumoniae* strains.

**Additional file 2: Table S2.** Extrachromosomal Genetic and resistome information of 14 colistin-resistant OXA-48-producing *K. pneumoniae* strains.

**Additional file 3: Figure S1.** A) The genetic environment of *bla*_OXA-48_ and *bla*_NDM-1_ in 14 colistin-resistant OXA-48-producing *K. pneumoniae* strains. *bla*_OXA-48_ was identified in all strains except for P26. The *lysR* and *bla*_OXA-48_ complex was found in all strains. No transposase element was detected surrounding the *lysR* and *bla*_OXA-48_ complex in P6, P7, P40, P42 and P43. Two *bla*_OXA-48_ with different genetic arrangement were found on P32 plasmid. In P36, P37, P38 and P44; the *lysR* and *bla*_OXA-48_ complex was flanked upstream by IS*91*, IS*4* and IS*110* family transposase genes, respectively. **B)** *bla*_NDM-1_ was detected only in P6 and P26. *bla*_NDM-1_ was flanked upsteram by bleomycin binding protein and *trpF* genes and downstream by the IS*91* family transposase.

## Data Availability

All generated data and materials are included in the text. This Whole Genome Shotgun project has been deposited at DDBJ/ENA/GenBank under the BioProject accession number PRJNA749021. The genomes described in this paper are version JAHYXE000000000, JAHYXD000000000, JAHYXC000000000, JAHYXB000000000, JAHYXA000000000, JAHYWZ000000000, JAHYWY000000000, JAHYWX000000000, JAHYWW000000000, JAHYWV000000000, JAHYXF000000000, JAHYWU000000000, JAHYWT000000000 and JAHYWS000000000.

## Abbreviations

AMR: Antimicrobial resistance
WGS: Whole genome sequencing
MDR: Multi-drug resistant
SAPs: Single amino acid polymorphysims
*mcr*: Plasmid-mediated colistin resistance
Co-CCRKp: Co-carbapenem colistin-resistant *K. pneumoniae*
CP-KP: Carbapenemase-producing *K. pneumoniae*
KPC: *K. pneumoniae* carbapenemase
CLSI: Clinical and laboratory standards institute
MIC: Minimal inhibitory concentration
CDSs: Coding sequences
CARD: Comprehensive antibiotic resistance database
ICE*K*p: Interactive conjugative element of *K. pneumoniae*
ABC: ATP-binding cassette
RND: Resistance-nodulation-cell division
NDM: New Delhi metallo-β-lactamase
ESBL: Extended spectrum β-lactamases
AmpC: Ambler class C T4SS: type IV secretion system
T4CP: Type IV coupling protein
ICU: Intensive care unit
IS: Insertion sequence
Inc: Incompatibility
