# Supplementary material for: Comparative genome analysis of colistin-resistant OXA-48-producing Klebsiellapneumoniae clinical strains isolated from two Iranian hospitals
Source: Ann Clin Microbiol Antimicrob. 2021 Oct 23;20:74. doi: 10.1186/s12941-021-00479-y (PMC8542297; doi:10.1186/s12941-021-00479-y)
Supplement: Supplementary file 1 — Additional file 1: Table S1. Genotypic, resistome profile and capsular and O-typing of 14 colistin-resistant OXA-48-producing K. pneumoniae strains. [file 12941_2021_479_MOESM1_ESM.docx]

**Table S1**. Genotypic, resistome profile and capsular and O-typing of 14 colistin-resistant OXA-48-producing K. pneumoniae strains.

| Strain | Chromosomal Genetic features | ST | AMR genes | Capsular K type | O Type |
| --- | --- | --- | --- | --- | --- |
| P6 | Total Length: 5,116,599 bp  GC Content: 57.5%  N50: 158,501  No. of CDSs: 4,801  No. of rRNA:3  No. of tRNA: 53 | ST147 | *oqxA, rmtC, mphA, aadA5, msbA, OmpA, OmpK37, KpnE, rsmA, KpnH, KpnG, emrR, adeF, eptB, fosA5, marA, lptD, arnT* and *h-NS* | K107 | O2v1 |
| P7 | Total Length: 5,151,344 bp  GC Content: 57.6%  N50: 167,625  No. of CDSs: 4,856  No. of rRNA: 5  No. of tRNA: 56 | ST147 | *oqxA, ampH, msbA, OmpA, OmpK37, KpnE, KpnF rsmA, KpnH, KpnG, emrR, adeF, eptB, fosA5, marA, crp, baeR, lptD, arntT, h-NS* and *crp* | K107 | O2v1 |
| P26 | Total Length: 5,212,103 bp  GC Content: 57.5%  N50: 323,312  No. of CDSs: 4,899  No. of rRNA: 3  No. of tRNA: 58 | ST147 | *bla* _SHV-11_, *ampH, msbA, OmpA, marA ,oqxA, OmpK37, KpnE, rsmA, KpnF, KpnH, KpnG, emrR, adeF, h-NS, eptB, fosA5, crp, baeR, lptD, arnT* and *ErmB* | K107 | O2v1 |
| P31 | Total Length: 5,261,999 bp  GC Content: 57.5%  N50: 153,291  No. of CDSs: 4,979  No. of rRNA: 3  No. of tRNA: 53 | ST11 | *bla*_SHV-11_, *oqxA, oqxB, KpnE, KpnF rsmA, KpnH, KpnG, emrR baeR, lptD, arnT, eptB, fosA6, marA, OmpK37, ompA msbA, h-NS, ampH, adeF* and *crp* | K15 | O4 |
| P32 | Total Length: 5,196,129 bp  GC Content: 57.6%  N50: 139,184  No. of CDSs: 4,916  No. of rRNA: 2  No. of tRNA: 51 | ST11 | *bla*_SHV-11_, *oqxA, oqxB, KpnF, KpnE, rsmA, KpnH, KpnG, emrR, baeR, lptD, arnT, eptB, fosA6, marA, OmpK37, msbA, h-NS, tet(B), tetR, rmtF, ampH* and *crp* | K15 | O4 |
| P33 | Total Length: 5,296,311 bp  GC Content: 57.5%  N50:152,800  No. of CDSs: 5,016  No. of rRNA:4  No. of tRNA:61 | ST11 | *bla*_SHV-11_, *oqxA, oqxB, KpnE, KpnF rsmA, KpnH, KpnG, emrR, baeR, lptD, arnT, eptB, fosA6, marA OmpK37, OmpA msbA, h-NS, ampH, adeF, tet(B), tetR* and *crp* | K20 | O4 |
| P35 | Total Length: 5,347,791 bp  GC Content: 57.5%  N50: 258,299  No. of CDSs: 5,071  No. of rRNA: 5  No. of tRNA: 63 | ST893 | *bla*_SHV-1_, *oqxA, OmpA, crp, KpnF, KpnE, rsmA, KpnH, KpnG, emrR, baeR, lptD, arnT, eptB, fosA6, marA, OmpK37, msbA, h-NS, ampH, crp* and *adeF* | K20 | O3/O3a |
| P36 | Total Length: 5,258,345 bp  GC Content: 57.5%  N50: 138,605  No. of CDSs: 4,971  No. of rRNA: 6  No. of tRNA: 66 | ST11 | *bla*_SHV-11_, *oqxA, oqxB, kpnE, kpnF, kpnH, KpnG, emrR, lptD, arnT, eptB, fosA6, marA, OmpK37, OmpA msbA, h-NS, rsmA, adeF, ampH* and *crp* | K15 | O4 |
| P37 | Total Length: 5,349,088 bp  GC Content: 57.2%  N50: 176,555  No. of CDSs: 5,077  No. of rRNA: 3  No. of tRNA: 56 | ST101 | *bla*_SHV-1_, *oqxA, KpnE, KpnE, KpnH, KpnG, emrR, baeR, lptD, arnT, eptB, fosA6, marA OmpK37, OmpA, h-NS, crp, rsmA, adeF, emrR* and *eptB* | K17 | O1v1 |
| P38 | Total Length: 4,941,238 bp  GC Content: 57.4%  N50: 215,119  No. of CDSs: 4,694  No. of rRNA: 3  No. of tRNA: 63 | ST893 | *bla*_SHV-1_, *KpnE, KpnF, baeR, lptD, arnT, eptB, fosA6, OmpK37, OmpA, msbA, h-NS, ampH* and *crp* | K20 | O3/O3a |
| P40 | Total Length: 5,386,704  GC Content: 57.3%  N50: 118,913  No. of CDSs: 5,118  No. of rRNA:5  No. of tRNA:61 | ST11 | *bla*_SHV-11_, *oqxA, oqxB, KpnE, rsmA, KpnH, KpnG, emrR, baeR, lptD, arnT, eptB, fosA6, marA, OmpK37, OmpA, msbA, adeF, tet(B), tetR* and *crp* | K15 | O4 |
| P42 | Total Length: 5,268,667  GC Content: 57.5%  N50: 153,054  No. of CDSs: 4,980  No. of rRNA: 4  No. of tRNA:60 | ST11 | *bla*_SHV-11_, *rmtF, crp, h-NS, oqxA, oqxB, KpnE, KpnH, KpnG, emrR, baeR, lptD, arnT, eptB, fosA6, marA, OmpK37, msbA, adeF, rsmA, ampH, OmpA, ampH* and *tetR* | K15 | O4 |
| P43 | Total length: 5,337,133 bp  GC content: 57.4%  N50: 200,728  No. of CDS:5,075  No. of rRNA: 3  No. of tRNA: 80 | ST11 | *bla*_SHV-11_, *crp,* *h-NS, oqxA, oqxB, KpnE, rsmA, KpnH, KpnG, emrR, baeR, lptD, arnT, eptB, fosA6, marA OmpK37, OmpA, msbA, adeF* and *rsmA* | K15 | O4 |
| P44 | Total Length: 5,303,649 bp  GC Content: 57.4%  N50: 149,160  No. of CDSs: 5,037  No. of rRNA: 6  No. of tRNA: 69 | ST11 | *bla*_SHV-11_, *crp, h-NS, oqxA, oqxB, KpnE,, KpnH, KpnG, emrR, baeR, lptD, arnT, eptB, fosA5, marA OmpK37, msbA, adeF, rsmA* and *msbA* | K15 | O4 |
