# Supplementary material for: Comparative genome analysis of colistin-resistant OXA-48-producing Klebsiellapneumoniae clinical strains isolated from two Iranian hospitals
Source: Ann Clin Microbiol Antimicrob. 2021 Oct 23;20:74. doi: 10.1186/s12941-021-00479-y (PMC8542297; doi:10.1186/s12941-021-00479-y)
Supplement: Supplementary file 2 — Additional file 2: Table S2. Extrachromosomal Genetic and resistome information of 14 colistin-resistant OXA-48-producing K. pneumoniae strains. [file 12941_2021_479_MOESM2_ESM.docx]

**Table S2**. Extrachromosomal Genetic and resistome information of 14 colistin-resistant OXA-48-producing K. pneumoniae strains.

| **Strain** | **Plasmids** | **Relaxase complex** | **Incompatibility** | **AMR genes** | **Integrons** | **Hypervirulence genes** |
| --- | --- | --- | --- | --- | --- | --- |
| P6 | Plasmid 1 | *oriT*: -  Relaxase: -  T4SS: -  T4CP: - | IncFIB(K)  IncHI1B | *bla*_CTX-M-15_, *bla*_TEM-1_, *bla*_NDM-1_, *aac(3)-IIe,* *aadA5 rmtC, mphA* and *dfrA17* | *intI1* | - |
|  | Plasmid 2 | *oriT*: +  Relaxase: +  T4SS: +  T4CP: + | IncL | *bla*_OXA-48_ | No | - |
|  | Plasmid 3 | *oriT*: +  Relaxase: -  T4SS: +  T4CP: - | IncFII | *bla*_OXA-1_, *ermB*, *crp* and *aac(6')-Ib-cr6* | *intI1* | - |
| P7 | Plasmid 1 | *oriT*: -  Relaxase: -  T4SS: +  T4CP: - | IncFII(Yp) | *bla*_OXA-48_ | No | - |
|  | Plasmid 2 | *oriT*: +  Relaxase: +  T4SS: +  T4CP: + | IncFIB  IncHI1B  IncL | *bla*_OXA-1_, *bla*_TEM-1_ and *aac(6')-Ib-cr6* | *intI1* | - |
| P26 | Plasmid 1 | *oriT*:-  Relaxase: -  T4SS: +  T4CP: + | IncFII (Yp) | *bla*_NDM-1_, *brp(MBL), rmtC* and *mphA* | *intI1* | - |
|  | Plasmid 2 | *oriT*: +  Relaxase: -  T4SS: -  T4CP: - | IncFIB(K)  IncHI1B | *bla*_CTX-M-15_, *qnrS1, aadA5, mphA, aac(3)-IIe, aph(6)-Id, dfrA17* and *aph(3'')-Ib* | *intI1* | - |
| P31 | Plasmid 1 | *oriT*: -  Relaxase: -  T4SS: +  T4CP: - | IncFIB  IncFII | - | No | - |
|  | Plasmid 2 | *oriT*: +  Relaxase: +  T4SS: +  T4CP: - | IncHI1B | *bla*_OXA-48_, *bla*_TEM-1_, *bla*_CTX-M-15_, *rsmA, aac(6')-Ib, aph(3'')-Ib, aph(6)-Id, tet(B), tetR, rmtF, arr-2* and *qnrS1* | *intI1* | - |
| P32 | Plasmid 1 | *oriT*: -  Relaxase: -  T4SS: +  T4CP: - | IncFIB  IncFII | *qnrS1, aph(6)-Id, aph(3'')-Ib,, aac(6')-Ib9,tet(B), tetR, rmtF* and *arr-2* | *intI1* | - |
|  | Plasmid 2 | *oriT*: +  Relaxase: +  T4SS: -  T4CP: - | Col440I | *bla*_OXA-48_, *bla*_TEM-1_ and *bla*_CTX-M-15_ | No | - |
| P33 | Plasmid 1 | *oriT*: +  Relaxase: +  T4SS: +  T4CP: - | IncFII | *bla*_OXA-48_, *qnrS1, aac(6')-Ib, tet(B), arr-2, tetR* and *catB3* | No | - |
|  | Plasmid 2 | *oriT*: -  Relaxase: -  T4SS: +  T4CP: - | IncFIB | - | No | - |
| P35 | Plasmid 1 | *oriT*: +  Relaxase: +  T4SS: +  T4CP: + | IncFII(K) | *qnrB1* and *tet(A)* | No | - |
|  | Plasmid 2 | *oriT*: +  Relaxase: +  T4SS: +  T4CP: + | IncL | *bla*_OXA-48_, *bla*_TEM-1_, *bla*_OXA-1_, *bla*_CTX-M-15_, *aac(6')-Ib-cr6, aph(6)-Id, catB3, aph(3'')-Ib, tet(A)* and *dfrA17* | *intI1* | - |
|  | Plasmid 3 | *oriT*: +  Relaxase: -  T4SS: -  T4CP: + | repB | - | No | *iutA, iucD, iucC, iucB* and *iucA* |
| P36 | Plasmid 1 | *oriT*: +  Relaxase: +  T4SS: +  T4CP: + | IncFII | *aac(6')-Ib9* and *arr-2* | *intI1* | - |
|  | Plasmid 2 | *oriT*: +  Relaxase: +  T4SS: +  T4CP: - | IncFIB | *bla*_CTX-M-15_, *bla*_OXA-48_, *bla*_TEM-1_  *qnrS1, aph(3'')-Ib, aph(6)-Id,* *tet(B)* and *tetR* | No | - |
| P37 | Plasmid 1 | *oriT*: +  Relaxase: +  T4SS: +  T4CP: + | IncFIB(K) | *mphA* | *intI1* | - |
|  | Plasmid 2 | *oriT*: +  Relaxase: -  T4SS: -  T4CP: - | IncFIB(K)  IncFIA(HI1) | - | No | - |
|  | Plasmid 3 | *oriT*: -  Relaxase: -  T4SS: +  T4CP: - | IncFII | *bla*_CTX-M-15_, *bla*_OXA-48_,  *dfrA14* and *ermB* | No | - |
| P38 | Plasmid 1 | *oriT*: +  Relaxase: -  T4SS: -  T4CP: - | repB | - | No | *iutA, iucD, iucC, iucB* and *iucA* |
|  | Plasmid 2 | *oriT*: -  Relaxase: -  T4SS: -  T4CP: + | IncFIB | - | No | - |
|  | Plasmid 3 | *oriT*: +  Relaxase: +  T4SS: +  T4CP: + | IncL | *bla*_CTX-M-15_, *bla*_TEM-1_, *bla*_OXA-48_, *bla*_OXA-1_, *aph(6)-Id, aph(3'')-Ib, qnrB1* and *dfrA17* | *intI1* | - |
| P40 | Plasmid 1 | *oriT*: +  Relaxase: +  T4SS: +  T4CP: + | IncFIB  IncFII | *bla*_TEM-1_, *qnrS1, rmtF*  *aph(3'')-Ib, aac(6')-Ib9* and *aph(6)-Id* | *intI1* | - |
|  | Plasmid 2 | *oriT*: -  Relaxase: -  T4SS: +  T4CP: - | IncFIB  IncHI1B | *aph(3'')-Ib* and *aph(6)-Id* | No | - |
|  | Plasmid 3 | *oriT*: +  Relaxase: +  T4SS: -  T4CP: + | IncFIB  Col440I | *bla*_OXA-48_, *bla*_CTX-M-15_ and *arr-2* | No | - |
| P42 | Plasmid 1 | *oriT*: -  Relaxase: -  T4SS: +  T4CP: - | IncFIB | *bla*_CTX-M-15_, *aph(3'')-Ib* and *aph(6)-Id* | *intI1* | - |
|  | Plasmid 2 | *oriT*: +  Relaxase: +  T4SS: +  T4CP: - | IncFII | *bla*_OXA-48_, *aac(6')-Ib, tet(B), tetR, rmtF* and *qnrS1* | No | - |
| P43 | Plasmid 1 | *oriT*: -  Relaxase: -  T4SS: -  T4CP: - | IncFIB | *qnrS1, tet(B)* and *tetR* | No | - |
|  | Plasmid 2 | *oriT*: +  Relaxase: +  T4SS: -  T4CP: - | Col440I | *bla*_OXA-48_ | No | - |
| P44 | Plasmid 1 | *oriT*: +  Relaxase: +  T4SS: -  T4CP: - | Col440I | *bla*_OXA-48_, *bla*_CTX-M-15_ and *bla*_TEM-1_ | No | - |
|  | Plasmid 2 | *oriT*: +  Relaxase: +  T4SS: +  T4CP: + | IncHI1B | *qnrS1, tet(B)* and *tetR* | No | - |
|  | plasmid 3 | *oriT*: +  Relaxase: +  T4SS: +  T4CP: + | IncFIB  IncFII | *rmtF, aph(3'')-Ib, aac(6')-Ib9* and *aph(6)-Id* | *intI1* | - |
