# Supplementary material for: Comparative genome analysis of colistin-resistant OXA-48-producing Klebsiellapneumoniae clinical strains isolated from two Iranian hospitals
Source: Ann Clin Microbiol Antimicrob. 2021 Oct 23;20:74. doi: 10.1186/s12941-021-00479-y (PMC8542297; doi:10.1186/s12941-021-00479-y)
Supplement: Supplementary file 3 — Additional file 3: Figure S1. A) The genetic environment of blaOXA-48 and blaNDM-1 in 14 colistin-resistant OXA-48-producing K. pneumoniae strains. blaOXA-48 was identified in all strains except for P26. The lysR and blaOXA-48 complex was found in all strains. No transposase element was detected surrounding the lysR and blaOXA-48 complex in P6, P7, P40, P42 and P43. Two blaOXA-48 with different genetic arrangement were found on P32 plasmid. In P36, P37, P38 and P44; the lysR and blaOXA-48 complex was flanked upstream by IS91, IS4 and IS110 family transposase genes, respectively. B) blaNDM-1 was detected only in P6 and P26. blaNDM-1 was flanked upsteram by bleomycin binding protein and trpF genes and downstream by the IS91 family transposase. [file 12941_2021_479_MOESM3_ESM.pptx]

## Slide 1
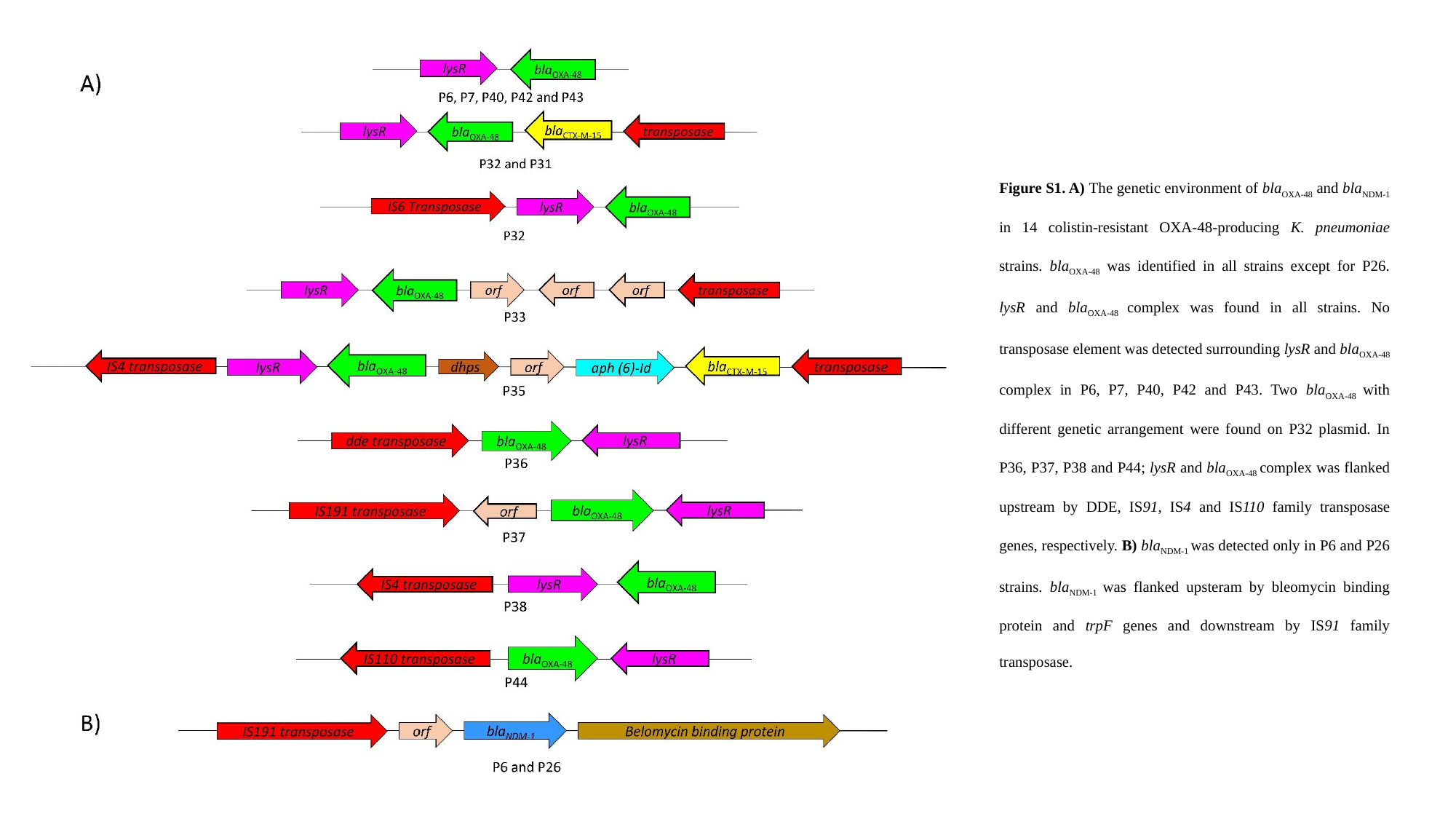

Figure S1. A) The genetic environment of blaOXA-48 and blaNDM-1 in 14 colistin-resistant OXA-48-producing K. pneumoniae strains. blaOXA-48 was identified in all strains except for P26. lysR and blaOXA-48 complex was found in all strains. No transposase element was detected surrounding lysR and blaOXA-48 complex in P6, P7, P40, P42 and P43. Two blaOXA-48 with different genetic arrangement were found on P32 plasmid. In P36, P37, P38 and P44; lysR and blaOXA-48 complex was flanked upstream by DDE, IS91, IS4 and IS110 family transposase genes, respectively. B) blaNDM-1 was detected only in P6 and P26 strains. blaNDM-1 was flanked upsteram by bleomycin binding protein and trpF genes and downstream by IS91 family transposase.
